# Parental Perceptions of Community and Professional Attitudes Toward Autism

**DOI:** 10.1007/s10803-024-06554-5

**Published:** 2024-10-07

**Authors:** Natalie Thayer, Christina Marsack-Topolewski, Kaitlyn Wilson

**Affiliations:** 1 Speech-language Pathology and Audiology, Towson University, Towson, MD USA; 2 College of Health and Human Services, School of Social Work, Eastern Michigan University, Ypsilanti, MI USA

**Keywords:** Autism, Professionals, Community members, Support, Professional attitudes

## Abstract

The purpose of this study was to examine parent perceptions of professional and community attitudes about autism through the lens of parenting their autistic children from birth through adulthood. Implications of this research may assist with future development and study of professional and community supports of individuals on the autism spectrum and their families. The study participants consisted of 51 parents who had an adult-aged child on the autism spectrum. One-on-one interviews were conducted with parents about their experiences raising a child with autism. Data were double-coded and analyzed using a qualitative, phenomenological approach to explore parents’ experiences with and perceptions of society members’ attitudes towards autism. Positive and negative themes were identified across parents’ perceptions of professionals and of community members. Themes regarding professional attitudes included cooperating with family requests, presuming competence of individuals with autism, complacency towards the needs of families and individuals with autism, and discrimination towards the individual with autism based on age or level of need. Themes regarding community member attitudes included valuing the strengths of individuals with autism, accommodating autistic differences, intolerance of autistic traits, and prejudice towards individuals with autism. Findings indicate that professional and community members presented with both positive and negative attitudes towards autism and individuals on the autism spectrum, with negative attitudes proving more prevalent in parent recollections. Results suggest a need for further research and related training to improve interactions with and support of individuals with autism and their families.

The autism spectrum consists of individuals born with brain variations that cause differences in communication, behavior, and learning compared to the general population (American Psychological Association, 2022). The “spectrum” of autism refers to the continuum of severity and heterogeneity of traits within these core differences. Current statistics demonstrate that the rate of autism diagnoses has increased among 8-year-old children in 11 sites in the United States from 1 in 150 children in the year 2000 to 1 in 36 in the year 2020 (Maenner et al., 2023). The cause of this increase in prevalence is uncertain, but theories suggest it may be due to increased awareness, expanded diagnostic criteria, and increased screening (Fombonne, 2020). Prevalence data is unavailable for children born before 1992; regardless, individuals on the autism spectrum were commonly institutionalized until the widespread process of deinstitutionalization began in the 1960s (Hollin, 2014). This change initiated the integration of individuals with autism into a general society that had little awareness or knowledge of autism.

In recent decades, the increase in autism diagnoses, as well as an increase in media representations, has increased society’s familiarity with autism and allowed more people to have personal relationships with individuals with autism. Knowing and spending time with an individual on the autism spectrum has been shown to have a positive effect on general society members’ attitudes towards autism (Dachez et al., 2015). People with limited experience or knowledge of autism may rely on media representations or anecdotes to gain information which may lead to preconceived notions about individuals with autism as a whole depending on how the autism spectrum is portrayed (Prochnow, 2014). According to Prochnow (2014), individuals with autism were described in the media in various ways, including “magical/savant, ‘different’/quirky, undiagnosed/unlabeled, and realistic portrayals” (p. 136). Media has historically failed to describe and portray autism on a spectrum, with minimal variance in symptomatology, behaviors, and characteristics (Prochnow, 2014). Even people with significant experience with individuals with autism (e.g., special education teachers) have been found to lack knowledge about autism (Gómez-Marí et al., 2021). This potential for misinformation and misrepresentation of autism could further isolate individuals with autism and their families and decrease social support.

Parents of children on the autism spectrum have a front-row seat to the complex nuances that accompany children with autism’s development, health, and interactions with the public, but their community often lacks the same knowledge. Autistic behaviors and emotional differences can cause stress for parents of individuals with autism (Huang et al., 2014), but additional parental stress stems from a lack of perceived social support which causes individuals with autism and their families to further avoid social situations (Devenish et al., 2020; Jones et al., 2021b). Jones et al. (2021b) reported that individuals with autism and their family members experienced social isolation, loss of friends and family relationships due to reactions to their or their family member’s autism, and unwillingness to leave the house due to potential negative reactions to their or their family member’s autism. Individuals with autism and their family members reported that they had avoided social environments including restaurants, cafes, concerts, sporting events, shops, and theaters because of environmental barriers (i.e., number of people or level of noise) and social barriers (i.e., worry about how people will respond to them or needing support in these places). Parents of children with autism may also perceive low social support due to concepts such as not wanting to burden others, stigma or lack of understanding about autism, or a sense that they are avoided due to their child’s challenges (Goedeke et al., 2019). The situation can be helped, however, because research indicates the addition of professional and community support has been linked to a decrease in stress (Devenish et al., 2020; Goedeke et al., 2019).

Families express a desire for consistent and effective collaboration with educators and other professionals, yet collaborative relationships can be stunted by professional attitudes (Anthony & Campbell, 2020). Effective parent-professional relationships were found to be based upon child-focused partnership factors, such as skills that helped children meet their goals and communication skills that supported the child and parent (Goldrich Eskow et al., 2018). Partnerships with culturally, linguistically, and economically diverse families were noted to benefit from the professional building strong rapport with the family, engaging in cultural reciprocity, and creating empowering environments for parents to feel comfortable sharing their priorities and goals (Pearson et al., 2019). Parent-professional relationship satisfaction resulted in parent-reported improvement in academic progress, intervention effectiveness, care-related stress levels, and family quality of life (Goldrich Eskow et al., 2018; Goedeke et al., 2019). A study by Werner (2011) assessed the attitudes of various students in health and human service professions programs (e.g., departments of social work, education, nursing, occupational therapy, and speech language pathology) and found that students perceived working with people with autism as difficult, challenging, and frustrating, yet rewarding, important, and an opportunity to develop personally and professionally. Although research exists on professionals’ attitudes towards autism (Werner, 2011), minimal research examines the parent perspective of professionals’ attitudes. Existing literature indicates professionals (e.g., teachers, employers) have varied attitudes towards autism, often moderated by knowledge, training, experience, and even gender (Buckley et al., 2021; Gómez-Marí et al., 2021). Professionals’ personal attitudes towards autism are often displayed through their actions and words, which are then observed and deciphered by families and individuals with autism themselves. This may contribute to a disconnect between an individual’s attitudes and how they are interpreted by the family. Interactions with professionals and the community are foundational building blocks for a child’s development, and therefore, they need to be positive and effective in order to meet the child’s and family’s needs.

To guide the field in future development and study of professional and community supports of individuals on the autism spectrum and their families, the purpose of this study was to examine parent input about their perceptions of professional and community members’ attitudes about autism. This study addressed the following research questions:

1. What are parents’ perceptions of community members’ attitudes towards autism?
2. What are parents’ perceptions of professionals’ attitudes towards autism?

## Method

This study used a qualitative phenomenological research method to examine the experiences of parental caregivers of adults with autism and their perceptions of professionals and community members regarding autism and people with autism. Phenomenological research is a qualitative approach that studies lived experiences from the individual’s perspective with the goal of describing the meaning of the experiences through the means of what was experienced and how it was experienced (Neubauer et al., 2019). This approach is used in health care research to help health care professionals learn from the experiences of others (Neubauer et al., 2019; Priest, 2002). Prior to the start of data collection, the Principal Investigator (PI) obtained Institutional Review Board (IRB #: 012615B3X) approval from Wayne State University.

### Author Positionality

As a qualitative research study, it is important to understand the authors’ positionality which acknowledges the authors’ backgrounds and worldviews and, therefore, the lens in which the data were analyzed. All three authors are Non-Hispanic White, female scholars. The first author has professional experience working with individuals with autism across the lifespan as a speech language pathologist and personal experience with close family members with special needs. The second author has experience as both a special education teacher and consultant in the public school system supporting adolescents with disabilities and their parents. Her research focuses on parental caregivers of adults with autism, focusing on national services, informal and formal support networks, caregiver burden and quality of life. The third author has research, teaching, and clinical experience with individuals with autism, with specific interests in improving the evidence-base surrounding interventions for individuals with autism across the lifespan. To avoid influencing the data, authors made efforts to bracket existing biases or assumptions before conducting data collection and analysis (e.g. the first author identified existing biases to refer to and examine while coding the data to ensure themes were aligned with data rather than personal experience).

### Recruitment

This study analyzes telephone interviews that were collected following a larger parent study consisting of an electronic survey completed by parents of adults with autism. Eligibility criteria for the survey included that parents were at least 50 years of age with an adult child (minimum of 18 years old) diagnosed with autism spectrum disorder (ASD) as stated by parent self-report. Age eligibility was determined with the intent to focus on older caregivers as the majority of published literature is directed on younger parental caregivers with younger children. Participants were recruited through formal organizations at the local, state, and national level, such as national autism support groups, as well as informal groups such as professional and community networks and informational events for parents of children with autism. Efforts were made to recruit from ethnically and racially diverse populations by specifically sending information to groups that serve diverse individuals. The study was shared through flyers, newsletters, websites, emails, and word of mouth. Recruitment included snowball sampling as participants shared the survey with other eligible peers.

In the larger study, an electronic survey was completed by 320 parents of adults with autism. The last question of the electronic survey asked parents if they would be willing to further discuss their experiences raising a child on the autism spectrum via a telephone interview. Of the 320 original respondents, 186 parents indicated interest in discussing their experiences raising a child with autism through adulthood in a one-on-one interview. From the 186 parents who indicated their interest, the PI randomly selected 51 parents who completed a semi-structured telephone interview. These parents were selected prior to examining any socio-demographic variables, including race and gender. The interviews lasted about one hour and were audio recorded with consent from the parents. Since the autism spectrum consists of a broad range of characteristics and support needs, the PI completed a large number of interviews to achieve a holistic representation of the varying experiences of parents.

### Sample

The initial study collected demographic information on the parents and their adult children with autism; however, no additional demographic information was collected from the interviewed parents to increase the respondents’ confidence in the confidentiality of their qualitative interviews. It is known that 46 of the 51 parents (90.2%) interviewed were mothers and 5 (9.8%) were fathers. Since the 51 interviewed parents participated in the larger study, it is assumed that their demographics are similar to the overall demographic information of the parent study. The parents’ ages ranged from 50 to above 70 years, with the majority between 50 and 59 years of age (*n* = 212, 66.3%), and most of them held higher education degrees (bachelor’s degrees [*n* = 100, 31.3%); master’s degrees [*n* = 90, 28.1%], and doctorate/professional degree [*n* = 33, 10.3%]). Although the PI attempted to recruit participants from organizations that were likely to contain racially and ethnically diverse parents, most of the participants were Non-Hispanic White (*n* = 289, 90.2%). Most of the parents were married (*n* = 252, 79.2%) and their annual family incomes ranged from less than US$20,000 to more than US$100,000 (see Table 1).

**Table 1:** Frequency distributions: parents’ demographics (*n* = 320)

Parents’ demographics					*n*	%
Parents’ age						
	50–59				212	66.3
	60–69				95	29.7
	70 and above				13	4.1
Gender of parent completing survey						
	Male				57	17.8
	Female				259	80.9
	Missing				4	1.3
Race and Ethnicity						
	African American/Black				5	1.6
	American Indian/Alaska Native				1	0.3
	Non-Hispanic White				289	90.2
	Hispanic				12	3.8
	Multiracial				6	1.9
	Other				5	1.6
	Missing				2	0.6
Marital status						
	Single, never married				6	1.9
	Married				252	78.8
	Divorced				41	12.8
	Widowed				11	3.4
	Cohabiting				2	0.6
	Separated				6	1.9
	Missing				2	0.6
Educational level of respondent						
	Less than high school				1	0.3
	High school/GED				10	3.1
	Some college				42	13.1
	Associate’s degree/technical school				44	13.8
	Bachelor’s degree				100	31.3
	Master’s degree				90	28.1
	Doctorate/professional degree				33	10.3
Annual family income						
	Less than US$20,000				14	4.4
	US$20,001-US$40,000				24	7.5
	US$40,001-US$60,000				42	13.1
	US$60,001-US$80,000				45	14.1
	US$80,001-US$100,000				37	11.6
	More than US$100,000				103	32.1
	I would prefer not to respond				48	15.0
	Missing				7	2.2

The survey also gathered demographic information of the parent respondents’ children on the autism spectrum. The adults with autism ranged in age from 18 to 44 years with most between 18 and 25 years (*n* = 190, 66.9%). Most interviewed parents reported their children on the spectrum were male (*n* = 41, 80.3%), with 10 parents indicating having a daughter on the spectrum (19.6%). Most of the adults with autism were reported to live with their parents (*n* = 248, 77.5%), with 16 (5.0%) living independently and 35 (10.9%) living independently with support. Some parents reported having two or more children on the autism spectrum or living with another chronic condition. In this case, the parent was asked to answer interview questions referencing their oldest child on the autism spectrum. Functional abilities of the adults with autism were determined by parent report regarding communication, behavior, and social competence levels. These levels varied from complete independence to needing a high degree of support (see Table 2).

**Table 2:** Frequency distributions: demographic characteristics of the adult child with autism (*n* = 320)

Adult children with autism’s demographics/characteristics							*n*		%
Age of adult child (M = 25.14, SD = 7.09)									
	18–20						71		25.0
	21–25						119		41.9
	26–30						52		18.3
	31–35						16		5.6
	36–40						11		3.9
	41 and above						15		5.3
Gender of adult child									
	Male						257		80.3
	Female						63		19.7
Living arrangement of adult child									
	Live independently						16		5.0
	Live independently with support						35		10.9
	Live in a group home						11		3.4
	Live with parents						248		77.5
	Live at college						6		1.9
	Other						4		1.3
Communication									
	Communicates a wide variety of topics meaningfully						124		38.7
	Communicates a limited range of topics meaningfully						112		35
	Communicates needs and wants in some meaningful way						29		9.1
	Communicates basic needs and wants						33		10.3
	Very little meaningful communication						17		5.3
	Uses device						4		1.3
	Other						1		0.3
Behavior									
	Typical, age appropriate adult behavior						14		4.4
	Requires minimal prompting, direction, or redirection						160		50
	Requires substantial prompting, direction, or redirection						108		33.8
	Engages in self-injurious behavior/dangerous behavior						19		5.9
	Requires 24-hour supervision to manage behavior						16		5.0
	Other						1		0.3
	Missing						2		0.6
Social Competence									
	Has a group of friends, goes out with others in the community						43		13.4
	Is part of a social group						88		27.5
	Would like friendships, but has no friends						73		22.8
	Is not able to make friends						32		10.0
	Socializes with family only						82		25.7
	Other						2		0.6

### Data Collection

One-on-one interviews were conducted by telephone over a span of two months, and all participants consented to the interview being audio recorded. After the interview, parents were mailed a US$20 gift card for participation in the study. The interview guide included nine open-ended questions that resulted in approximately one-hour long interviews.

Interview questions were cultivated to examine the caregiving experiences of aging parents of adults with autism. Questions included topics such as types of support received, satisfaction with support received, and the rewards and challenges of caring for an adult on the autism spectrum. Participants discussed current and past experiences through these questions. Although the questions did not directly ask the parents about their perceptions regarding attitudes of community members and professionals pertaining to autism, their responses provided information on their perceptions of this topic. Prior to the interviews, eight professionals (i.e., school social worker, teacher consultants, special education teacher, school psychologist, speech pathologist, and transition coordinator) experienced with ASD professionally, and some personally, reviewed the interview questions and provided constructive feedback to ensure the validity of the interview questions. Feedback from professionals was used to refine the interview questions to achieve a high relevance of data obtained from the interviews.

### Data Analysis

The completed interviews were professionally transcribed, then reviewed by the PI to ensure accuracy of the interview transcripts. Transcripts were redacted of any identifying information of the parent or their child to assure participant anonymity. The phenomenological approach to data analysis follows the procedural steps outlined in Polkinghorne (1989). The line-by-line coding process involves dividing the responses into units, identifying the meaning of the unit to assign a theme, then grouping the meaning-based themes to describe participants’ experiences. To increase the reliability of the qualitative coding, two researchers independently coded all data relevant to the attitudes of professionals and community members regarding autism. First, the two researchers met and discussed key concepts and definitions of community members, professionals, and attitudes. Community members were defined as neighbors, friends, extended family members, and individuals in professions that do not provide specialty services to individuals with autism. Professionals were defined as people who performed an autism-related service. Attitudes were defined as a settled way of thinking or feeling about someone or something that is reflected in a person’s behavior. The researchers coded units of the data into meaning-based themes, then sorted the themes into positive and negative attitudes of community members or professionals. The researchers compared coding results and were in consensus on 90% of positive and negative themes that emerged from the data. After discussing the final 10% of themes, the researchers were in accord with all themes.

## Results

Although interview questions did not directly address society’s attitudes towards autism, parents openly shared their experiences on this topic. After analyzing the data, researchers found that parents experienced both positive and negative attitudes towards autism across community and professional groups. This is expected due to external factors such as location, demographic information, and personal attributes of individuals. Multiple parents reported conflicting experiences with professionals and professional organizations that varied by state, county, or individual service providers. Both positive and negative themes are important to highlight to bring forward areas of strength and areas for improvement in community and professional interactions with individuals with autism and their families.

Two positive and two negative themes emerged pertaining to community member attitudes. These included the positive attitudes of valuing the strengths of individuals with autism and accommodating autistic differences and, conversely, the negative attitudes of intolerance of autistic traits and prejudice towards individuals with autism. Additionally, two positive and two negative themes emerged pertaining to professional attitudes. This included the positive attitudes of cooperating with family requests and presuming competence of individuals with autism and, contrarily, the negative attitudes of complacency towards the needs of families and individuals with autism and discrimination towards the individual with autism based on age or level of need. To present the findings of the data per our research questions, both positive and negative themes from community members and professionals will be examined and described with quotes from the parent interviews illustrating their perceptions. Pseudonyms are used to maintain the confidentiality of the parents.

### Community Members

In the present study, community members were defined as neighbors, friends, extended family members, and individuals in professions that do not provide specialty services to individuals with autism (e.g., police officers and store clerks). Attitudes were defined as a way of thinking or feeling about autism and related individuals or experiences that manifests in a person’s behavior. Researchers have compiled and reported the most prominent themes from the data.

#### Valuing Strengths of Individuals with Autism

Many parents in the study reported community members with supportive attitudes toward their child with autism and their family. They described people, mainly adults, taking special interest in their child throughout their lifespan to support their development socially, academically, vocationally, and personally. In these situations, community members valued the child’s strengths which was shown through community member behaviors of group inclusion, vocational opportunities, mentorship, and friendship. One mother, Sharon, discussed how her adult child’s college roommates appreciated his strengths leading to social group inclusion and personal growth. She stated, “He has great roommates. He’s doing all these activities, and he’s doing physical activity for the first time in his life. His roommates got him off his sugar addiction. He’s happy now. He wasn’t this happy before.”

Another way community members supported an individual with autism’s strengths was by treating the individual as capable. This occurred on Jane’s son’s high school swim team about which she reported,

The swim coach said, “As long as he follows the rules like everybody else, we have no problems.” He lettered twice in high school and went on to swim in college. It was the work ethic that the coaches loved. As the coach put it to me, “He’s not a natural swimmer, but seeing him bloom, blossom, and become his own man was worth it.”

Although the coach noted that swimming was not a natural strength of the child, the child’s strong work ethic provided a foundation to learn and gain proficiency in the sport. The coach’s appreciation of the child’s strengths allowed him the opportunity to develop physical skills, be included in a team, and find a hobby that he continued to enjoy beyond high school.

Findings highlight many supportive attitudes from people in the individual with autism’s workplace from customers, other employees, and supervisors. One mother, Michelle, discusses feedback from her son’s supervisor at his new university research job. She shared, “[His supervisor] said she was thrilled with his work because he’s so focused. He doesn’t waste time talking to anybody. It takes seeing him work and getting past the initial interviewing difficulties to see what he’s able to do. She sees that. […] He’s found shortcuts for her, he’s created a new database to make her work easier, and all the things you just hope for.” Michelle went on to share the effect of the supervisor’s support on her son. She stated, “Now, he comes back home and his head is held high, his shoulders are back, and he’s feeling good about himself. This is the first time I’ve seen him feeling the least bit confident about himself.” The supervisor valued the child’s strengths leading to a well-matched vocational opportunity that benefitted the child’s career advancement and self-confidence. Had the supervisor not valued the child’s strengths, she may have placed weight on the child’s interviewing difficulties and not given him the chance to show his job-specific abilities.

#### Accommodating Autistic Differences

Another positive theme found in parent interviews included community members accommodating individuals with autism’s differences. Community members showed an attitude of accommodation for the individual with autism’s differences through behaviors of inclusive programming, sensory-trained staff, listening to the voices of people with autism, and helping the individual reach their goals.

An example of a community group with accommodating attitudes towards neurodiverse differences is highlighted by Abigail who explained,

Within our synagogue, we created these Friday night services, so everyone can feel comfortable. It’s really comforting to see families, whose kids can’t sit still or might need to talk or walk around, feel comfortable. They don’t feel like somebody’s looking at them or somebody’s going to ask them to leave.

These accommodations benefit individuals with autism and others, such as families with young children, to allow everyone to share in the traditions of their community. Another form of accommodating attitudes reported in parent interviews involved staff being trained to work with sensory-sensitive individuals. Examples included dentists and hair stylists who used sensory-sensitive techniques as well as court staff who provided accommodations for individuals with sensory needs.

Coworkers and family friends were shown to have accommodating attitudes about autism and their peers on the autism spectrum by listening to the needs of the individual with autism and helping them reach their goals. Jamie’s daughter, Katie, was diagnosed on the spectrum as a young adult. Her mother shared that the label helped her daughter self-identify in the workplace and share what she experiences. Jamie stated that Katie communicates,

“My brain works a little differently,” or, “If you see me going into this situation-.” She can tell people, “I’m starting to have a meltdown, please don’t touch me.” Because at her past job everybody wanted to touch her, but in a meltdown she can’t accept that. So being able to say, “If I have a meltdown, this is what it looks like and here’s what to do,” was a phenomenal help for her and for them.

Jamie’s daughter advocated for her needs and described situations in which she needs specific supports. Her coworkers’ accommodating attitudes in conjunction with direct instruction from Katie allowed her a safe and productive work environment. Other examples of community members showing accommodating attitudes by listening to the needs of the individual on the autism spectrum and helping them reach their goals included siblings’ friends playing videos games with the sibling with autism, parents’ friends understanding the child with autism’s challenges and helping them practice their social skills, and special driving programs designed to help individuals with differences gain independence through driving.

#### Intolerance of Autism and Related Experiences

Intolerance on different levels was found in the attitudes of community members. For some people, they did not want to spend the extra time and energy to learn about autism or help the family raise their child on the autism spectrum. When talking about his extended family and friends, one father, Michael, explained,

They don’t understand, and they don’t want to understand. When we try to give them information or try to share reading material or anything like that, they don’t bother to read what we give them. They don’t bother to educate themselves about this issue. I don’t, I can’t even begin to explain why that is, but they don’t choose to learn about it, they don’t choose to understand.

Michael’s experience was common across participants, and it caused many parents to report feelings of isolation within their communities.

Another form of intolerance was recognizing the strengths of the individual with autism, but not accepting their traits that were different from those of neurotypical people. This was commonly found in employers who appreciated the individual’s work ethic or skill set, but did not tolerate their slower learning style or different social skills. One example of this comes from a mother, Marcia, who knows her adult child with autism lacks neurotypical social skills. Marcia shared,

I had one gal that I have known since [my son] was a baby. She used to have an ice cream shop and he’d stop in there when he would go for his walks. She saw me one day and said, ‘He is really good with computers, and I would have hired him to do a web page but…’. I can’t think of what her reasoning was, but he couldn’t really communicate with her, and I think she couldn’t really communicate with him. I was shocked that she said it, ‘I would have hired him but’. So that’s kind of the attitude around here.

A complete form of intolerance was externalized by exclusion of the individual with autism and their family. Exclusion of the individual with autism was discussed from same-age peers, extended family members, and parents’ friends. Besides social exclusion of the individual with autism themselves, exclusion takes many forms for the parents and families. This consists of not being invited to events, being invited to events conditionally if the child with autism does not come, losing friends who do not relate to their different child-rearing experiences, difficulty finding romantic partners, and people not willing to help with childcare. All of these types of exclusion cause social isolation for parents and families, shame around their family’s situation, and added stress from the lack of assistance. One mother highlighted how she lost her job due to her employer’s intolerance of her family needs. When asked by the interviewer if she had run out of paid time off allowance, she shared,

I’d been there long enough that I had the time [to take off]. It’s just that being able to do the job and then the pressures of the people that don’t understand what it’s like having someone that you have to take care of. They just don’t understand, you’re not gonna be sitting at lunch smiling, laughing, and talking about recipes and things like that. You’re gonna be in your office on your cell phone with one of twelve people on your son’s medical and support team making appointments, trying to transition him back into the high school, and then having to take him back out of the high school. So they just didn’t understand that my cell phone calls weren’t personal things to have fun. They were a necessity to coordinate transitions that we’ve had to make over the last few years.

Reaching professionals during their work day is an essential need for parents of individuals on the autism spectrum as they coordinate medical appointments, set and attend educational meetings, and handle unplanned challenges. Employers’ intolerance of the needs involved in raising a child with autism can lead to a loss of job for the parent and, therefore, loss of income for the family.

#### Prejudice Towards Individuals with Autism

Nearly all of the parent interviews explored the theme of prejudiced attitudes, preconceived opinions that were not based on actual experiences. Community members were often found to lack understanding about autism and replace this understanding gap with prejudice. This was shown in one quote by mother, Emily, who has a child with autism and a daughter who is a behavioral therapist for individuals with autism. Emily shared,

You know what the hardest part is? When you go out in public, how can people still stare at you? Or how come my daughter works […] She’s a behavioral therapist and she took her kids out on a field trip. They went into a restaurant and one of the kids was so excited he ordered very loudly. It wasn’t bad. He was just loud. And the guy behind the counter asked them to leave.

The employee who asked the group to leave interpreted the loud behavior as negative when the child was expressing his excitement. Lack of community education about people with autism and their traits further perpetuates these misinterpretations.

Community members also harbored preconceived ideas about autism and individuals with autism which caused negative interactions with family members. These ideas included that people with autism were “the center of attention”, “too old to not take care of themselves”, “not behaving”, and “had a bad attitude”. People with prejudiced attitudes towards autism attributed the individuals’ food aversions, unexpected behaviors, and absence of neurotypical social skills with poor parenting. These preconceived ideas further developed into fear of the child on the autism spectrum and hate towards them and their family. One father, Brady, disclosed his son, “was bullied in school to the point where some kid beat him up, broke his nose in four places by kicking him in the face with his shoe. I yanked him out of school because the school could not protect him.”

Fear of autism and prejudiced attitudes towards individuals with autism come to a dangerous intersection when it comes to the police. Many parents discussed events where community members witnessed behavior outbursts by individuals with autism and called the police. One mother, Sherriah, discussed a personal situation involving her daughter and recalled,

There have been times when she’s kind of lost it in stores. One time she was upset with me about something and was pulling my scarf and yelling. People around us were looking and wondering what’s going on. So I just worry about that, when we’re out in public, I’m just afraid one time it isn’t going to go well and people aren’t going to understand. You read all these surveys, I mean all these articles and studies and stuff, saying the people who are shot most, you know, by police, and it’s people who have disabilities because they don’t know how to handle them. Well, what happens if I’m not there, or I’m there and they don’t care?

Sherriah’s fears were not extraordinary. Multiple parents shared experiences of their children with autism being confronted by the police, and the police did not have the knowledge of or consideration for autistic behaviors and traits. The most prominent traits that police officers did not understand included stimulatory behaviors, social skill differences, and concrete mindset. When asked what could improve the lives of people with autism and their families, one mother commented,

Making sure that local law enforcement or police officers really understand ASD.

One day [my daughter] was driving her car and […] it used to be a police car. [My husband] got it cheap and all of the markings were off of it, but it still kind of looked like a police car. So she’s driving down the road and a cop’s following her. She just keeps going and finally a second cop comes up and she pulls off the side of the road. The first cop says, “Why didn’t you stop?” and she said, “Cause I didn’t do anything wrong.” And that’s the way an Asperger’s mind thinks. And the cop is like, “When a cop follows you, you have to stop,” and she’s like, “But I didn’t do anything wrong.” Well, pretty soon his backup is there, and they’re deciding whether to arrest her or not. They’re like, “Why are you driving a police car?” And she’s like, “It’s not a police car. This is my daddy’s car. He gave it to me.” Finally, the second one talked the first one into not pursuing anything. […] So [what I want is] for police and law enforcement officials to understand what Asperger’s is and not to hurt these kids or these adults that have these attributes.

### Professionals

In this study, professionals were defined as people who performed an autism-related service. This includes educators, medical professionals, service providers (e.g., speech language pathologists, occupational therapists, psychologists, etc.), and government organizations (e.g., Department of Rehabilitation). Parents reported both positive and negative experiences with professionals and their organizations. In some instances, parents reported varied experiences between state, county, or individual service providers. This study reports on all experiences regarding perceived attitudes to develop the following themes.

#### Presuming Competence of Individuals with Autism

An important positive theme was professionals presuming competence of the individual with autism. This competence extended to friendships, work, self-regulation, leadership, and independence. Professionals who presumed competence pushed the child with autism and their family to new experiences in order to achieve a greater potential. One example of this included a family’s excellent public school experience with their son, Spencer, who has autism. His mother shared, “He had some great teachers that saw him for who he was, made some accommodations. I mean so many of them were just like, Spencer, talk to me about this topic. Help me understand how much you understand about it. They weren’t worried that he couldn’t answer ‘X’ number of questions in 15 minutes.” The educators’ accommodations showed that they presumed Spencer’s competence in the learned material, but understood that traditional testing would not appropriately reflect his knowledge. Appropriate and individualized ways for individuals with autism to present their skills and knowledge gives professionals and families an accurate representation of the individuals’ strengths. This can lead to further beliefs of competence in the individual with autism and helps professionals determine additional opportunities that may fit the individual well.

Another example of presumed competence leading to opportunities for the individual with autism includes a mother discussing the role of a professional life coach working with individuals with autism. She shared, “[The life coach] specialized in solving problems one on one for kids with Asperger’s. She was real connected in the town, and, by golly, she found them jobs that would match their skills.” This compares to community attitudes described in this paper when community members valued the strengths of an individual with autism and gave them an opportunity to learn and grow in a job or team.

#### Cooperative with Family and Requests

Many professionals were reported to have a cooperative attitude with the family and the individual with autism by collaborating with the family and providing helpful services. Families reported that helpful services were individualized, accessible financially, and treated the individual as a whole. Professionals were also perceived as cooperative when they listened to the needs of the family and the autistic community when creating their goals and treatment plans.

An important way a cooperative attitude was shown through professionals’ behavior included professionals collaborating with the family to reach the individual with autism’s goals. When discussing collaboration, parents often noted that exponential progress towards their children’s goals was made when compared to times without collaboration. One mother explains,

The most important component is that we work together as a team. I have her cell phone number and she’s got mine, so there’s a great line of communication. We reinforce whatever they do in school. If we didn’t reinforce what they did, and if they didn’t reinforce what we did, a lot of these behavioral issues would still be there.

Collaboration also led to feelings of well-being and success in parents. Another mother describes, “When he was in preschool, his teacher came to my house and we agreed on [a behavior plan]. Now it is consistent, the teacher and the mom are saying the exact same thing. How extraordinary is that.” A cooperative attitude in the form of collaboration, especially from educators and service providers, was found to increase family satisfaction with services, trust within the team, and mastery of goals for the child.

One example of professionals providing helpful services includes Casey’s experience with transition programs for her young adult daughter on the autism spectrum. She specifically recalled the supports of the College Internship Program (CIP). She shared,

They have a curriculum of [many] levels of executive functioning where they deal with hygiene, banking, independent living […] She lived in an apartment with two other girls, and the center was right in the same complex so the transition was magnificent. We just had all these experts.

When asked why Casey perceived the model as very effective for her daughter’s transition from school to young adult life, Casey stated,

It’s systematic, and it treats the individual as a whole instead of little pieces. Plus they had enough staffing to be able to have the right go-to people. However, the greatest gift was, [my daughter], she had a community. She had a community of peers, a circle of friends, people that were like her.

This individualized support that treated her daughter as a whole was beneficial in providing her daughter with necessary life skills to boost her strengths and address her specific areas of need.

An example of financially accessible, helpful services due to strong cooperation includes Jeff’s positive experience with his local mental health agency. He shared,

The biggest thing I would say is for any parent to get on board with the mental health agency and know that you have resources through them for your adult child. We didn’t know any of this until our hand was forced into it because we had to file guardianship, and you can’t do that without a mental health agency. So, they’ve been instrumental in helping us gain services for [my son] and that includes SSI, staffing, and Medicaid. The mental health agency, they have a lot of resources. And so, if I didn’t have them, it would be difficult.

Jeff’s positive experience was due to the professional organization’s cooperation with the family to provide helpful services that are financially accessible, individualized to their needs, and include different supports to treat many needs of the family and individual. Many other parents did not report positive experiences with professional organizations due to lack of cooperation with the family and neglect of the family’s needs.

#### Complacency Towards Needs of Families and Individuals with Autism

Despite the many ways professionals were supportive, parents found many professionals to display a complacent attitude towards themselves and their child with autism. Complacency involved apathy with regard to family needs and professionals’ satisfaction with their own abilities that prevented them from learning more or working harder. One demonstration of professionals’ complacent attitudes was a refusal to consider parent requests. Parents disclosed requests for appropriate services, such as respite care workers that were physically capable of caring for their child, IEP goals that aligned with the child’s greatest needs, and vocational training that is applicable to the job market. Many parents discussed needing to advocate for their child to receive the services that would benefit the child most. When Karen, a mother who attributed her son’s high quality of services to her education and willingness to advocate, remarked, “I think that he gets good psychiatric care, but if we were not involved with the intense management of the details in his life, he would get inadequate psychiatric care.” The need to advocate for appropriate services was present across environments, from education, medicine, and therapeutic services, which bred distrust and dissatisfaction with professional organizations and individuals. When parents were unsuccessful with their efforts, many families involved legal representatives to assist in advocating for services. With service quality and availability reportedly based on the amount of time, money, and energy a parent can spend advocating, professionals create service disparities between active, affluent parents and parents who do not have the resources or knowledge to request adequate services from complacent professionals.

Professional organizations, such as Community Mental Health, Department of Rehabilitation, and respite care staffing agencies, were also found to display complacent attitudes by not following through on family requests for assistance with questions, eligibility status, and documentation requirements. One mother of a son on the autism spectrum, Shellie, describes when the manager of her son’s favorite store offered him employment. Shellie recalled,

I went to the school and said if they could help do whatever paperwork then I pretty much had a job lined up for my son. They wanted to know who it was with and that’s as far as it went. So nothing happened with that. Then, I called the guy from Department of Rehab, and I told him that I was working on for a job for my son, but I need to go through the channels. He said he couldn’t help me, but he said, “If you got it lined up, come back and tell me”. They were no help. I battled with everyone.

Shellie’s story was echoed in other interviews, where parents contacted various organizations for assistance and were unable to find help. This lack of follow through resulted in children with autism and their families missing out on many opportunities and beneficial services.

Many parents recounted professionals lacking understanding in autism diagnoses, autistic traits, and autistic strengths. Due to the generation the parents in this study raised their children, a lack of understanding could be attributed to the lower rate of autism in society and lower awareness of autism as a diagnosis. Examples of this include parent accounts of misdiagnoses and late diagnoses. This phenomenon displayed a complacent attitude as professionals’ satisfaction with their own abilities prevented them from learning more about the diagnosis and treatment of autism. Many parents recalled their children being diagnosed with bipolar disorder or other psychiatric conditions, and the medical professionals medicated their children as such. This often caused physical and mental distress for the child and caused mistrust in the medical system for the parent. One parent, whose son was diagnosed with bipolar disorder before being diagnosed on the autism spectrum, recalls “He was being treated for bipolar disorder and put on all these medications. He started to have hallucinations from them. We were like, ‘Okay, something’s wrong here.’ And he wasn’t any better behaved. He was on medications for about two years.” Complacent attitudes are represented in this quote by the continuation of the treatment and medication for bipolar disorder despite no improvements to the child’s areas of need. Another parent, Trisha, whose daughter wasn’t diagnosed on the autism spectrum until twenty-seven, shared, “I had her hospitalized at ten, but they were not diagnosing autism in verbal children at that time.” Because of this late diagnosis, Trisha’s daughter did not receive therapeutic services while in school, and she believed she “was crazy” because she thought differently than the rest of her family.

#### Discrimination Due to Age or Level of Need

The final negative theme found from parent interviews includes professionals having a discriminatory attitude towards people with autism due to their age or level of need. Many participants recalled the lack of services for young adults with autism after completing grade school. Parents reported that there are very few services for adults with special needs, referring to the transition as a “big black hole” or a “big abyss”. Parents commented that most existing programs do not solve the problems that arise as a child transitions to an adult. Gillian, a mother who has moved states to find better services for her child with autism, states,

There are going to be so many kids diagnosed on the spectrum in the coming years. I don’t think our culture has even begun to address this. Then, when they turn 18 and the school just dumps them out into the community, or wherever it is they dump them up to, it’s going to be a disaster, it really, really is. There are going to be so many struggling with this issue.

One parent reported that their local university speech pathology clinic would not accept adult clients with autism because they “make less progress”. By training student therapists to only feel success when their client makes progress, society perpetuates the lack of services for adults with special needs.

Other parents noted conflicting experiences with vocational skill training programs. Vocational skill training programs that were reported to be highly effective were reported to have a high financial cost which limited who could benefit from the services. Other parents described that the appropriate services their child needed were extremely expensive leading to financial instability and even bankruptcy. Training programs that exhibited discriminatory attitudes were shown to either not accept individuals because they had too many support needs or not accept individuals because they presented with a high IQ and “did not need services”. The language of being too “high functioning” or “low functioning” to benefit from services was present across interviews and across settings. The terms “high functioning” and “low functioning” attempt to describe the degree in which an individual with autism is impaired in daily living skills by assessing the individual’s cognition level, or IQ. These terms are outdated as recent research has shown that IQ is a weak predictor of adaptive behavior in individuals with autism (Alvares et al., 2020). In addition, the terms are misleading as they may disqualify individuals from receiving services. Current alternatives to describing a person’s level of functioning include clinical specifiers found in the DSM-5 (i.e., with or without intellectual impairment; with or without language impairment)(APA, 2013), stating the individual’s support needs (i.e., requires substantial communication support), or identifying specific strengths and weaknesses to guide appropriate services (i.e., strong communication skills; vocational skill needs)(Alvares et al., 2020). Caroline, a mother of an adult child with autism with high IQ and high communication skills, shared “He’s very frustrated. Sometimes, I have seen him in tears saying, ‘I’m too broken to function properly and I’m not broken enough to qualify for help.’”.

## Discussion

The purpose of this qualitative, phenomenological study was to identify the perceived attitudes of professionals and other community members towards autism through the lens of parents of adults on the autism spectrum. During their interviews, parents shared both positive and negative experiences involving interactions with professionals and community members. Through community member interactions, positive themes of valuing the strengths of individuals with autism and accommodating their differences were found as well as negative themes of intolerance of autistic traits and prejudice towards individuals with autism. Through analysis of interactions with professionals, positive themes of cooperating with family requests and presuming competence of individuals with autism were found as well as negative themes of complacency towards the needs of families and individuals with autism and discrimination towards the individual with autism based on age or level of need. Since the participants in this study have adult children, their frequent experiences with prejudice and discrimination could be due to the years they raised their children when autism was not as common of a diagnosis. On the other hand, one parent remarked, “I don’t know if you would call it a backlash, but a lot of people, I don’t think they think Autism Spectrum is real. You know, it’s kind of like, ‘Yeah, that’s the newest trendy diagnosis.’”.

This study suggests that parental actions were affected by community and professional attitudes. Families developed distrust in the medical system, the education system, therapeutic service providers, and government organizations. This is consistent with other research citing specific challenges such as shortcomings in diagnosis, inefficient services, and organizational challenges in autism services (Gholipour et al., 2022). Additionally, parents developed feelings of shame, guilt, and isolation in their communities. The experiences shared in this paper are consistent with other research including parents feeling blamed for their child with autism’s behavior (Neely-Barnes et al., 2011) and feeling rejected and excluded from their communities due to stigma (Kinnear, 2015). Some parental actions were not of their own volition as they experienced loss of jobs, bankruptcy, and legal involvement due to employer and professional attitudes. These secondary effects of raising a child with autism are preliminary findings that warrant further research.

Support is essential for parent, child, and family quality of life. Positive community themes of valuing the strengths of individuals with autism and accommodating autistic differences provided individuals with autism and their families with social support. With increased support and accommodation in a community, children with autism are shown to increase community involvement and participation, and parents report reduced feelings of isolation and increased coping abilities (Devenish et al., 2020). Helpful accommodation of differences includes minimal barriers for involvement, high levels of helpfulness, and increased availability of resources. However, themes also included community members’ intolerance of autistic traits. Research has found that individuals with autism and their families often withdraw from social situations due to lack of social support, such as negative reactions in the community due to intolerance of autistic behaviors or mannerisms (Jones et al., 2021b), therefore increasing parental stress (Devenish et al., 2020). The community theme of prejudice is consistent with previous literature in which non-autistic adults perceived discrimination against people with autism, yet when surveyed about their attitudes towards people with autism, discrimination was found in their own responses (Jones et al., 2021b).

Throughout the lifespan, individuals with autism experience social rejection and isolation at high rates (Underhill et al., 2019), and exclusion from the community increases after the individual with autism transitions out of formal education (Taylor et al., 2017). Results imply that this exclusion may be a result of intolerance of autistic behaviors and prejudice towards the individual with autism. This could be due to the traditional approach of increasing the communication and “social skills” of people with autism by teaching them to conform to the norms of general society, rather than teaching general society about the differences in autistic communication. We need to transition from attempting to “fix” individuals with autism towards addressing attitudinal barriers in the community. Evidence to support this reverse teaching model includes the general public showing increased positive attitudes towards autism after spending time with a person with autism because the personal experience allowed them to develop their own perspectives on autism and dispel any preconceived notions (Kuzminski et al., 2019; Dachez et al., 2015). Additional trainings that have proved successful in increasing general society members’ understanding and acceptance of autism include short educational videos and presentations about autism that included both factual information and first-person narratives from individuals with autism (Gillespie-Lynch et al., 2022; Jenks et al., 2023; Jones et al., 2021a). These videos were found to improve explicit biases including higher expectation of autistic abilities, positive impressions of adults with autism, lower stigma about autism, and greater social interest in people with autism. Some participants noted the benefit of learning both the challenges that come with autism and the positive characteristics that balance the barriers. To improve autism education and inclusion, efforts should address training non-autistic people not only for awareness of autism diagnosis and its presentation, but knowledge and first-person accounts from the voices of individuals on the autism spectrum themselves.

Additional training is also needed at the level of law enforcement personnel to decrease prejudice towards individuals with autism and improve interactions between police and citizens with autism. Although a majority of police officers report interacting with individuals with autism, research suggests they lack knowledge about autism and how to handle situations involving individuals with autism (Christiansen et al., 2023). Training programs should be developed with individuals with autism who can provide lived experiences and insight into where interactions go wrong as well as recommendations on how they can be improved (Salerno-Ferraro & Schuller, 2020). Advocacy and support from the community are important to spearhead these training efforts.

To act on themes of cooperating with family requests, professionals providing autism-related care should operate within a family-centered approach and increase parent education. Best practice for fostering a family-centered approach includes a unified interprofessional team, parent education on the range of intervention approaches, and individualized recommendations for the needs of the child and family (Sandbank et al., 2020). Parent education should include the range of evidence-based intervention approaches as well as information on complex topics discussed by participants such as advocacy, health care, transition services, and employment. Resources can be found online or at local Parent Training and Information Centers (PTIs) and Community Parent Resource Centers (CPRCs) (Anthony & Campbell, 2020). Parents expressed a desire for consistent and effective collaboration with and between educators and other professionals, yet they often experienced disconnection between service providers. Given the present study’s findings, professionals should enhance communication with the family as well as other members of the child’s interprofessional team. Parent-educator and parent-professional collaboration increases family quality of life (Hsiao et al., 2017) and gives parents a sense of self-efficacy in managing their child’s goals and progress (Anthony & Campbell, 2020).

Themes of professionals’ complacency towards the needs of families and individuals with autism and community members’ intolerance of autistic traits were demonstrated through a lack of accommodation for and inclusion of the individual with autism and their family. A lack of accommodation is found across research with the following examples providing a brief depiction. Although there is ample evidence to support the types of educational accommodations that benefit people with autism, less than 35% of individuals with autism reported receiving these accommodations (Jones et al., 2021b). This continues into the workplace where intolerance for autistic differences deprives people with autism of necessary supports to be successful.

To decrease discrimination due to lack of knowledge or understanding, professionals need to gain understanding of autistic traits, strengths, and needs. This will aid professionals in providing beneficial and relevant services to individuals across the autism spectrum. Research shows that training programs can increase professionals’ knowledge about autism, perceived competency in autism interventions, and perceived competency in parent-professional collaboration (Griffiths et al., 2023). Successful training programs may include current information on autism and neurodevelopmental differences, best practices in interventions and behavioral strategies in the school and home, collaboration and communication in special education, and mental health of individuals with autism.

### Study Limitations

This study has limitations that may be addressed in future studies. The sample of the study was predominantly Caucasian female parents who had higher income and education levels when compared to the general public. Future studies should analyze the similarities and differences in perceptions for fathers and parents of different cultural or socioeconomic backgrounds. Many parents discussed professionals and their service quality or quantity varying by location. While this study created a comprehensive image of professionals in the United States, further research may inquire about specific regions, states, or counties. Another limitation is the study was based on parent interviews which did not take into account the individual with autism’s perspective on the matter. Additional research should examine the perceptions of younger parents of children and adolescents on the autism spectrum to understand their perceptions regarding community members and professionals. In addition, further research should explore individuals with autism’s perceptions of society’s attitudes, how their own perceptions shape their experiences, and how societal attitudes shape their experiences.

## Conclusion

This study is novel in its focus on parents who were at least 50 years of age with adult children on the autism spectrum. These parents’ perceptions and experiences as they raised an individual with autism through childhood to adulthood are needed to understand the challenges they encounter before, during, and after aging out of school-based support. The findings in this study indicate that community members and professionals presented with both positive and negative attitudes towards autism and individuals with autism, with negative attitudes proving more prevalent in parents’ recollections. Themes regarding community member attitudes included valuing the strengths of individuals with autism, accommodating autistic differences, intolerance of autistic traits, and prejudice towards individuals with autism. Themes regarding professional attitudes included cooperating with family requests, presuming competence of individuals with autism, complacency towards the needs of families and individuals with autism, and discrimination towards the individual with autism based on age or level of need. As the prevalence of individuals on the autism spectrum continues to increase, a need exists for further research on the attitudes of society and related training to improve society’s interaction with and support of individuals with autism and their families.
